# Disentangling social isolation, loneliness, and later-life cognitive function for older adults in the United States: evidence from causal inference modeling

**DOI:** 10.1093/geronb/gbaf254

**Published:** 2025-12-16

**Authors:** Jo Mhairi Hale, Angelo Lorenti, Solveig A Cunningham

**Affiliations:** School of Geography and Sustainable Development, University of St Andrews, St Andrews, Scotland; Max Planck Institute for Demographic Research, Rostock, Germany; Max Planck Institute for Demographic Research, Rostock, Germany; Max Planck-University of Helsinki Center for Social Inequalities in Population Health, Rostock, Germany; Hubert Department of Global Health, Rollins School of Public Health, Emory University, Atlanta, Georgia, United States; Netherlands Interdisciplinary Demographic Institute, den Haag, The Netherlands; (Social Sciences Section)

**Keywords:** Dementia, Health, Well-being, Life course, g-Formula

## Abstract

**Objectives:**

Older adults are at high risk of the negative health impacts of social isolation and loneliness. One of those possible negative health impacts is Alzheimer’s disease, a leading cause of death for adults in the United States and many high-income countries. Taking a life course perspective, we explore whether there is a direct causal effect of social isolation on later-life trajectories of cognitive function, the extent to which any effect of social isolation on cognitive impairment operates indirectly through loneliness, who may be most vulnerable, and the potential efficacy of a statistical intervention for those living alone.

**Methods:**

We use a counterfactual approach, the g-formula, with the U.S. Health and Retirement Study, analyzing data from 30,421 individuals with 137,653 observations across 2004–2018.

**Results:**

We find a consistent pattern of social isolation having a detrimental direct causal effect on cognitive function, with only 6% of this effect operating through loneliness. Reducing social isolation has a protective effect on cognitive function for all subpopulations regardless of gender, race/ethnicity, and educational level, with only minor differences among social categories. Our statistical intervention shows that targeting social isolation in those living alone may be one viable public health strategy for protecting against cognitive decline.

**Discussion:**

Our results suggest that addressing social isolation—and, by extension, its effects on health—requires both a broad understanding of its heterogeneous impacts on the general population and a nuanced approach to targeting public health interventions where they can be most effective.

Often framed around the rise of social media usage and the COVID-19 pandemic, there has been a recent spate of research on social isolation and loneliness, especially focused on adolescents and older adults. In the United States, even prior to the pandemic, about a quarter of older individuals were socially isolated (Cudjoe et al., 2020), spurring concerns about the health impacts of isolation on the older population (Umberson & Donnelly, 2023; Umberson et al., 2022). Likewise, loneliness has been recognized as a major public health issue in high-income countries, including the United States, the United Kingdom, Sweden, Australia, Germany, and Japan. These governments’ concerns are informed by evidence of the detrimental health impacts of social isolation and loneliness, ranging from low subjective well-being to multimorbidity and shorter life expectancy (Freilich et al., 2024; Hajek et al., 2023; Holt-Lunstad, 2018; Hong et al., 2023; Steptoe et al., 2013). Population aging has provoked a focus on pathological cognitive decline as another possible consequence of social isolation and loneliness, as a growing share of the population is living more years at ages with a higher risk of cognitive impairment.

In the United States, Alzheimer’s disease, the most common cause of pathological cognitive decline, already afflicts an estimated 6.9 million people (Alzheimer’s Association, 2024). As an incurable leading cause of death for which efficacious treatments remain elusive, researchers often focus on modifiable risk factors. There is some associational evidence suggesting that greater isolation and loneliness, ostensibly modifiable risk factors, are correlated with worse cognitive function (Fan et al., 2024; Kim & Hwang, 2024; Kuiper et al., 2015; Lim et al., 2020; Raymo & Wang, 2022; Yu et al., 2021; Zheng, 2021). However, as is often the case in health research, a significant challenge lies in the interdependent nature of social isolation, loneliness, and cognitive function that can lead to a dynamic relationship.

To address this challenge, we use causal inference modeling (the parametric g-formula) and nationally representative, population-based data to estimate the effect of social isolation and loneliness on later-life cognitive function. A benefit of the g-formula is that it provides estimates of the total effect (TE). This is a “population-averaged effect” that is useful from a public health perspective because it enables the simulation of interventions. As such, we are able to estimate what we hypothesize to be the direct effect of social isolation on cognitive function; its indirect effect as it operates through loneliness; differences in vulnerability across gender, race, ethnicity, educational attainment, and household composition; and the impact of a statistical intervention reducing social isolation for those living alone.

## Background

### Defining social isolation and loneliness

The assumption that social isolation leads to loneliness has meant that research often considers them jointly or even uses them interchangeably. In summarizing the literature here, we retain the authors’ terminology in following these assumptions. However, we will distinguish between isolation and loneliness, as the next step in advancing this research is to examine them as independent and interacting forces (Bu et al., 2021; Fawaz & Mira, 2023; Mansfield et al., 2024; Steptoe et al., 2013). As Hong and colleagues (2023) encourage, we must “characterize the shared and unique ways in which social isolation and loneliness influence health and well-being” (p. 9). Indeed, crafting efficacious interventions depends on understanding (1) the underlying risk factors for both social isolation and loneliness, (2) the extent to which loneliness is a mechanism connecting isolation with poor cognitive function, (3) the modifiable factors that might disrupt the link between isolation and cognitive function, and (4) the subpopulations most at risk. To that end, we will clarify our definitions.

Social isolation is the *objective* measure of the degree to which an individual is isolated from other humans. Research on social isolation has been constrained by the varying metrics available in datasets; Cornwell and Waite (2009) note this as “both a blessing and a curse” (p. i39). For example, social isolation has been measured as an index that includes all or some of the following: partnership status, household composition, social network size, frequency of contact with family and/or friends, employment status, religious participation, and engagement in social activities (Berkman & Syme, 1979; Cornwell & Waite, 2009; Shankar et al., 2013; Taylor et al., 2019; Zavaleta et al., 2017). Based on two such indices, researchers found the prevalence of social isolation amongst older U.S. residents as 21% to 25% (Crowe et al., 2021; Cudjoe et al., 2020). Consistent with the broader well-being literature, Cudjoe and colleagues found that men, those living alone, those with lower levels of education, and those with lower incomes were more likely to be isolated. Evidence is mixed on the racial/ethnic disparities in social isolation (Taylor et al., 2019; Umberson & Donnelly, 2023), as can be anticipated from inconsistent approaches to measurement (Cornwell & Waite, 2009).

Loneliness, on the contrary, is a *subjective* construct of the feeling that there is a discrepancy between an individual’s desired and actual social connections (Perlman & Peplau, 1981). In survey research, loneliness is often measured using single-item self-reports of frequency of loneliness. However, there are a number of commonly used scales, including the de Jong-Gierveld Loneliness Scale (de Jong-Gierveld, 1987) and the UCLA Loneliness Scale (Russell et al., 1978). The single-item self-report is highly correlated with the UCLA scale and has been found to be appropriate for use with older populations (Luchetti et al., 2020), although it has lower validity for some groups, such as men (Raymo & Wang, 2022). Prevalence is estimated to range from 15% to one-third of older U.S. residents, depending on measurement (Raymo & Wang, 2022; Solway et al., 2019). Women and those unemployed, living alone, with poorer health, and with lower socioeconomic status are more likely to report loneliness than their counterparts on both self-reports and the UCLA scale (Dahlberg et al., 2022). Racial/ethnic differences in loneliness remain understudied (Taylor et al., 2023), thus inconclusive (Barjaková et al., 2023).

Previous studies have indicated associations between social isolation, loneliness, and health. A meta-analysis of 70 prospective studies with almost 3.5 million participants in Europe, Asia, and North America shows approximately 30% higher risk of earlier mortality (even excluding suicide and accidents) for those objectively socially isolated and/or subjectively lonely, even after accounting for age, gender, and baseline health status (Holt-Lunstad et al., 2015). In addition, loneliness carries a substantial nonfatal health burden; it is associated with lower subjective well-being, multimorbidity, and more frequent physician visits (Lim et al., 2020). As described above, much research has not adequately distinguished between social isolation and loneliness (Taylor et al., 2023); however, there are some notable exceptions, especially recently (Bu et al., 2021; Fawaz & Mira, 2023; Kobayashi & Steptoe, 2018; Steptoe et al., 2013). For example, U.S.-based (Hong et al., 2023) and European-based (Fawaz & Mira, 2023; Steptoe et al., 2013) longitudinal studies reported that social isolation was associated with a greater increase in mortality risk than loneliness. Steptoe and colleagues (2013) also found that the association between isolation and mortality was unchanged by controls for loneliness.

### The entanglement of social isolation, loneliness, and cognitive function across the life course

A life course perspective on how the accumulation of disadvantages affects cognitive health complicates traditional regression analyses, as similar risk factors lead to social isolation, loneliness, and cognitive impairment—all of which are likely to be in a feedback loop. For example, early-life socioeconomic status (SES) and stress exposure are predictive of later-life ­isolation, loneliness, and cognitive health (Chen et al., 2022; Cudjoe et al., 2020; Dahlberg et al., 2022; Hale, 2017). Furthermore, childhood SES is also associated with other elements of one’s life between early exposure and later life, such as educational attainment, which is the strongest modifiable predictor of later-life cognitive impairment (Leggett et al., 2019).

Complicating modeling, childhood SES, and educational attainment predict both labor force participation and cognitive function (Hale et al., 2021), as well as isolation and loneliness (Morrish & Medina-Lara, 2021; Wiertz & Lim, 2019). All of these exposures are also associated with eventual partnership status, which is also predictive of cognitive function: Being partnered is associated with a lower risk of cognitive decline (Sharma et al., 2024). Partnership is also a strong predictor of social isolation and loneliness. Many of these pathways are bidirectional, for example, cognitive decline can be both caused by and cause people to be socially isolated and lonely.

Thus far, we have focused on modifiable risk factors, but one of the most important social predictors of later-life cognitive function is race/ethnicity, with Black and Latinx individuals experiencing significantly higher risk of cognitive impairment than Whites (e.g., Zhang et al., 2016). This is partially driven by disparities in educational and occupational opportunities (Reskin, 2012), but is also likely related to exposure to the stresses of discrimination (Das, 2013). Associations between gender and cognitive function are less clear, especially for earlier cohorts, where educational and occupational opportunities for women were limited. As noted above, evidence for race/ethnicity, gender, and loneliness is inconclusive (Barjaková et al., 2023). Some findings suggest that identifying as Black, Latinx, and/or a woman is positively associated with loneliness (Raymo & Wang, 2022), but, importantly, negatively associated with social isolation (Cudjoe et al., 2020).

In sum, the pathway to both the outcome (cognitive function) and the main predictors of interest (social isolation and loneliness) involves an entire life course of compounding advantages and disadvantages that are interconnected.

### Disentangling life course pathways to social isolation, loneliness, and cognitive function

This article will address three key reasons why social isolation and loneliness research has been inconclusive with regard to cognitive impairment. First, the research often conflates the concepts of social isolation and loneliness, which are aligned but distinct. Second, the predictive power of quantitative approaches has often been limited by reliance on cross-sectional data, shorter follow-up periods, or associational methods. Studying loneliness as a personality trait disregards two types of individual factors: internal (e.g., age) and external (e.g., partnership status) that shift over a life course. Third, and relatedly, analyses also most often treat social isolation and loneliness as point-in-time outcomes, neglecting life course risk pathways (Holt-Lunstad, 2018; Umberson & Donnelly, 2023).

One reason that these life course risk factors have not been disentangled is the methodological challenges. The three primary issues are the following: (1) pathways between predictor and outcome are likely dynamic and bidirectional, for example, social isolation affects cognitive function and vice versa; (2) exposures and time-varying mediators may intersect, for example, social isolation’s effect on cognitive function may depend on the extent to which that isolation causes loneliness; and (3) mediator-outcome confounding, for example, loneliness affects social isolation and cognitive function and is affected by both. The parametric g-formula that we use, unlike traditional regression models, does not deliver biased estimates in the face of these issues (Buckley et al., 2015; Keil et al., 2014; Naimi et al., 2017).

## Method

To estimate the effect of social isolation and loneliness on later-life cognitive function, accounting for time-variant and invariant sociodemographic risk factors, we use the Health and Retirement Study (HRS) (1992–ongoing). The HRS is a longitudinal, nationally representative, biennial survey of U.S. residents over age 50 and their spouses, regardless of age (grant number NIA U01AG009740) (HRS, 2022). Some of our measures are derived from RAND Version Q of the HRS (*RAND HRS Data, Version 2018-V2*, RAND Center for the Study of Aging, 2022), and we also draw variables from “HRS C 1992-2019” of the Gateway to Global Aging Harmonized Data (Center for Economic and Social Research, 2023). We use data from all waves for retrospective data, but our analysis focuses on data from the 2004 to 2018 waves, in which loneliness, social isolation, and selected cognitive function measures were assessed consistently.

We select a subsample consisting of main and spouse respondents aged 50 to 94 years. Given the focus on cognitive function and self-assessed loneliness, we exclude individuals who required a proxy respondent. Missingness on loneliness and depression is primarily due to survey design (e.g., respondents with proxy informants were not asked TICs or CES-D questions), and there is less than 1% missing on other covariates. Respondents only have to participate in two consecutive waves to be included. The final analytical sample includes 137,653 observations from 30,421 individuals.

### Key study measures

#### Outcome: cognitive function

The HRS’s modified version of the Telephone Interview for Cognitive Status (TICS-m) was designed to be sensitive to pathological cognitive decline and minimize ceiling and floor effects (Fong et al., 2009). We include the University of Michigan Survey Research Center’s imputed values (McCammon et al., 2019). From the TICS-m, following a large body of literature (Crimmins et al., 2011; Langa et al., 2023), we extract a subset of questions that are reflective of neurophysiological health: immediate (0–10 points) and delayed word recall (0–10 points), serial 7 s (backward counting from 100 by sevens) (0–5 points), and counting backward from twenty (0–2 points). The range is 0 to 27, where higher values represent better cognitive function.

#### Primary exposure: social isolation index 2004–2018

Over its course, the HRS has included various indicators of social isolation, some of which are asked in each wave and some only in leave-behind questionnaires that are completed by half the sample every other wave, leaving a 4-year gap between measurement of predictor and outcome. For this analysis, where we seek greater precision between exposure and the outcome of cognitive function, we use only measures available biennially. Compared with research that uses a 4-year follow-up (e.g., Hong et al., 2023), this does limit the indicators that can be included. However, from the available indicators, we were able to generate a social isolation index (SII) adapted from the psychometrically evaluated Berkman–Syme Index (Berkman & Syme, 1979), which, though conceived decades ago, remains fairly consistent with contemporary literature (e.g., Hong et al., 2023).

Our SII (see Table 1) takes into consideration three components of engagement. One is *sociability* measured through partnership/marital status, time spent with family and friends, and likely barriers to sociability. The second component is *church group membership*, measured through self-reported frequency of religious participation. The third component is *membership in community organizations*, measured through self-reports of how many hours respondents spend volunteering per year. The SII range is from 0 to 8, categorized as ≤5 (the median) = less isolated, or 6 to 8 = more isolated.

**Table 1.** Social isolation index created from Health and Retirement Survey indicators and adapted from the Berkman–Syme index (Berkman & Syme, 1979).

Engagement component	Less isolated (=0)	More isolated (=1)
**Sociability and its barriers**	Partnered	Not partnered
	Provides 100+ hr of grandchild care per year	<100 hr of grandchild care per year (or no grandchildren)
	100+ hr of helping friends per year	<100 hr of helping friends
	Uses email	Does not use email
	No issues with mobility	Mobility issues
	No hearing impairment	Hearing impaired
**Church membership**	Religious participation several times a month or more	Yearly or no religious participation
**Community organizations**	100+ hr of volunteering per year	<100 hr of volunteering

*Note.* Each indicator equals 1 point (range, 0–8).

Unlike the Berkman–Syme Index (1979), we analyze household composition separately from the SII so that in the last step of the analysis, we can establish the effect of a simulated intervention on a targeted subpopulation—those living alone.

#### Mediator: loneliness

As part of the Center for Epidemiological Studies–Depression (CES-D) scale, respondents report whether they felt lonely in the past week (0 = not lonely, 1 = lonely). This indicator for loneliness has been measured consistently across the longest timespan and is administered every wave.

#### Covariates

Time-invariant covariates include *birth cohort*, which follows the HRS cohort structure (AHEAD 1919–1923, Children of the Depression Era 1924–1930, HRS 1931–1941, War-babies 1942–1947, Early Baby-boomers 1948–1953, Mid Baby-boomers 1954–1959, Late Baby-boomers 1960–1965, and Early Generation X 1966–1971). We control for *Region of Interview* as part of HRS’s sampling strategy (Northeast, Midwest, South, West, Other). *Race/Ethnicity* combines self-reported race and ethnicity: non-Hispanic White, African American/Black Hispanic, non-Black Hispanic, and non-Hispanic Other (henceforth White, Black, Latinx, Other). HRS reports a binary *Gender* variable (women, men). *Childhood SES* is a self-report of the family’s financial situation when the respondent was younger than age 16 years (poor, average, well-off). *Educational attainment* is categorized as less than high school/general equivalency diploma (GED), high school diploma, some college/associate’s, or at least a bachelor’s degree. *Wealth* is based on a RAND-generated measure of household wealth that includes assets and debts. We create wave-specific wealth quintiles and then average them over the study period to get a time-invariant measure meant to be reflective more of later-life SES than annual income, which is less meaningful for this age range where a large proportion of respondents are retired.

Time-variant covariates include *age* in completed years, modeled through natural cubic splines to allow for the nonlinear association between age and cognitive function. *Household composition* is classified as alone, with partner, with children, with partner and children, or other. *Labor force participation* is categorized as working part-time/full-time, retired, unemployed, disabled, or not in the labor force (NILF). *Comorbidities* combines self-reported diagnoses of stroke, diabetes, heart condition, and/or high blood pressure/hypertension. *Depression* is measured as a single, binary item (yes, no) from the CES-D, a self-report of whether the respondent felt depressed in the last week. We select this over the full CES-D summary measure because our analysis also incorporates the loneliness item from the same scale.

### Analytical strategy

To deal with the methodological intricacies posed by a dynamic relationship with feedback between treatment, mediator(s), and outcome, we use a counterfactual framework based on the parametric g-formula (Robins, 1986). The g-formula is a statistical technique that extends standardization to address the challenges associated with time-varying exposure and confounding (Wang & Arah, 2015). The advantages of this method include not only the generation of hypothetical scenarios but also the yielding of estimates that are not affected by overcontrol bias and collider-stratification bias. Although this method does not ensure causal conclusions, it generates valuable population-level insights that are crucial for evaluating plausible policy interventions.

#### Basic steps of the g-formula

**Figure 1.**
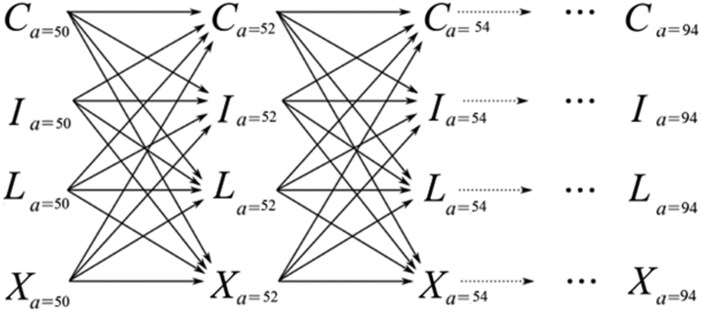
Directed acyclic graph showing the theoretically derived cross-lagged model.

We implement the g-formula in four steps:

Step 1: Define the Directed Acyclic Graph (DAG) that portrays the underlying data generation, addressing potential feedback between cognitive function, social isolation, loneliness, and other time-varying characteristics by using a cross-lagged design (Figure 1).

Step 2: Simulate the data following the DAG and reproducing the observed data. The simulated data that resemble the observed data are called the natural course, as it reflects the concept of an individual following a “natural course of events” (Supplementary Figures 1–3, see online supplementary material).

Step 3: Intervene on the simulated data to reduce social isolation.

Step 4: Contrast the intervention scenario (Step 3) to the natural course (Step 2) to evaluate the causal effect of reducing social isolation on cognitive function.

We provide a detailed description of each step, the model assumptions, and the methodological approach in Supplementary Methods (see online supplementary material).

#### Steps for moderation, mediation, and dynamic intervention

The TE yields an estimate of the impact of reducing social isolation on cognitive function. This effect may vary across population subgroups, influenced by underlying mechanisms. To delve deeper into how social isolation differentially affects cognitive functioning, we start with a moderation analysis. This analysis estimates the TE within specific racial/ethnic groups and by educational attainment.

Following that, we undertake a mediation analysis to assess the extent to which social isolation’s effect on cognitive function is mediated through loneliness. We determine the direct effect on cognitive function of reducing social isolation by using simulations that mirror the intervention scenario designed for the TE. In these simulations, the mediator of interest—loneliness—is maintained at its natural course level to ensure that the statistical intervention does not influence the mediators, thereby isolating the portion of the intervention effect that does not operate through loneliness. The indirect effect—the portion that does operate via loneliness—is then calculated by subtracting the direct effect from the TE. This calculation helps us understand the extent to which the benefits of reducing social isolation are mediated by its relationship with loneliness.

After evaluating the effect of the intervention (main analysis) and its heterogeneity (moderation), and evaluating the indirect effects to assess the extent to which loneliness mediates the effect of isolation on cognition (mediation), we simulate a dynamic intervention targeting only individuals living alone, a time-varying characteristic.

The parametric g-formula allows the estimation of counterfactual outcomes under different interventions, including those conditional on covariates (i.e., dynamic treatment rules), provided the identification assumptions—exchangeability, positivity, and consistency—are met (Hernán & Robins, 2020; Westreich et al., 2012) (Supplementary Methods, see online supplementary material). In this case, living alone is not considered an exposure, but rather a condition that defines the intervention rule. A targeted intervention such as this aims to identify the extent to which it would be successful on a hypothetically vulnerable population.

## Results

### Descriptive statistics

As predicted from the literature, the correlation between isolation and loneliness (across 30,421 individuals with 137,653 person-waves) is not very strong. Of those who report being lonely, only 55% fall into the category of more isolated, and about 26% of older adults report feeling not lonely despite being more isolated (Table 2). There are differences in social isolation in the analytical sample by sociodemographic factors: Older individuals, Latinx, and Black individuals appear to be more isolated, as do those who had lower childhood SES, those who report lower educational attainment, and those in the lowest wealth quintile. Those living with others, working, with fewer comorbidities, and not depressed are less isolated than their counterparts.

**Table 2.** Key descriptive statistics of analytical sample by social isolation index score, Health and Retirement Study 2004–2018.

Variable	Social isolation index		
	Less isolated %	Isolated %	Sample size (person-wave)
**Total**	69%	31%	137,653
**Loneliness**			
**Not lonely**	74%	26%	114,049
**Lonely**	46%	55%	23,604
**Age in years, *M***	65	72	
**Race/ethnicity**			
**White**	71%	29%	91,556
**Black**	66%	34%	24,646
**Latinx**	63%	37%	16,985
**Other**	74%	26%	4,466
**Gender**			
**Men**	71%	29%	56,787
**Women**	68%	32%	80,866
**Childhood socioeconomic status**			
**Poor**	62%	38%	41,267
**Average**	71%	29%	85,901
**Wealthier**	77%	23%	10,485
**Education**			
**Less than high school/GED**	55%	45%	43,296
**High school**	69%	31%	55,398
**Some college/associate’s degree**	79%	21%	7,881
**College+**	86%	14%	31,078
**Wealth quintile**			
**Lowest**	52%	48%	43,866
**Second**	67%	33%	32,029
**Third**	79%	21%	24,471
**Fourth**	83%	17%	26,631
**Highest**	87%	13%	10,656
**Household composition**			
**Live alone**	44%	56%	33,195
**Partner**	83%	17%	58,063
**Children**	48%	52%	9,711
**Partner and children**	85%	15%	16,532
**Other**	65%	35%	20,152
**Labor force status**			
**Full-time/part-time work**	84%	16%	41,969
**Retired (or partly)**	62%	38%	81,378
**Unemployed**	76%	24%	3,157
** Not in the labor force and disabled**	60%	40%	11,149

*Note.* See online supplementary material  Supplementary Table 1 for the full version of the table.

### Analytical results

To assess the effect of reduced social isolation on cognitive functioning, we compare the age curves describing the patterns of cognitive function in the natural course with the intervention scenario. The difference between the curves is the TE of the intervention on social isolation (shifting from greater isolation, 6 to 8 on the SII scale, to less isolation, ≤5) on cognitive function. Although the insulative effect varies by age, the intervention is associated with higher cognitive function by about 0.19 (95% confidence interval [CI] = [0.13, 0.24]) (men: 0.15, 95% CI = [0.09, 0.22] and women: 0.21, 95% CI = [0.14, 0.28]), averaging across the age trajectory. While this may seem modest on a scale of 0 to 27, the average cognitive decline across ages 50 to 94 is from a score of approximately 16 to 7, so only about 9 points. Also, we are examining a policy-relevant shift of SII, not from high isolation to zero isolation. In short, reducing social isolation is protective against cognitive decline, net of loneliness and controls.

**Figure 2.**
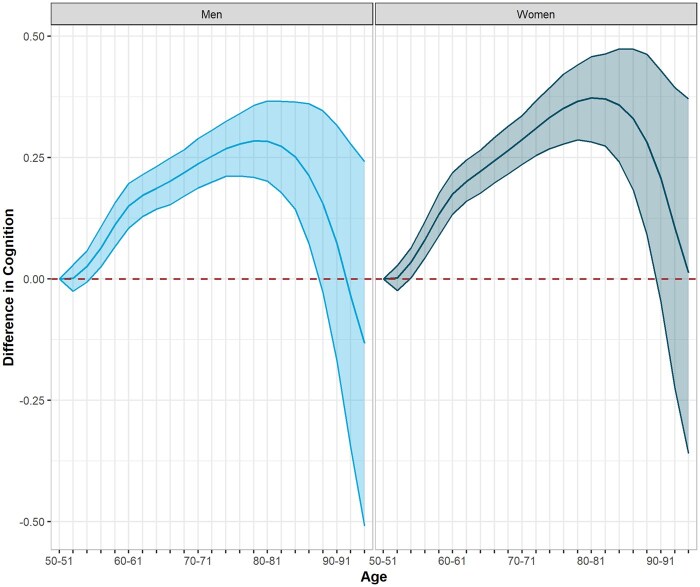
Average treatment effect on the treated by age and gender of how reducing social isolation is protective against cognitive decline.

Figure 2 shows, by age and gender, that the average treatment effect on the treated (ATT—the effect only on those who had higher social isolation) of reducing social isolation is cumulative until older ages, where it ceases to be above zero. The effect is slightly higher than the TE, for all 0.20 (95% CI = [0.14, 0.25]) (men: 0.16, 95% CI = [0.10, 0.23]) and women: 0.22, 95% CI = [0.16, 0.29]).

#### Moderation analysis: Race/ethnicity and educational level

**Figure 3.**
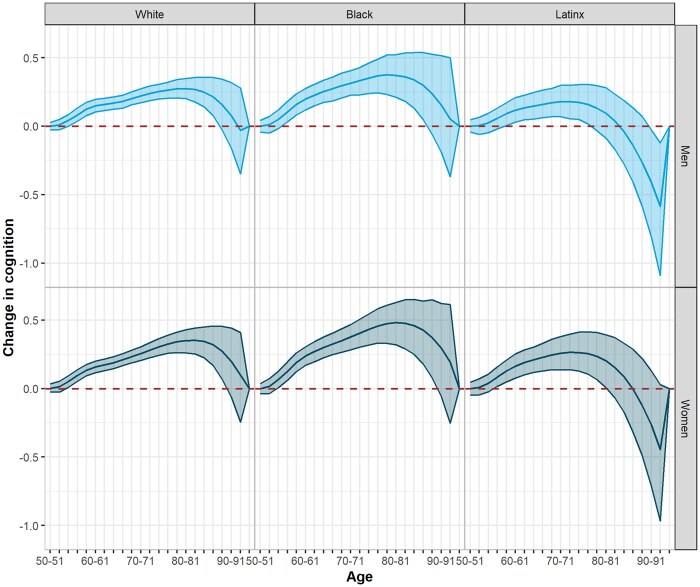
Average treatment effect on the treated showing the effect of reducing social isolation on trajectories of cognitive function across age by race/ethnicity.

The effect of the intervention may be heterogeneous across subpopulations, and such heterogeneity typically has substantive and theoretical implications. Moderation occurs when, for example, the treatment effect for the subpopulation who identify as Black is different from that for the subpopulation who identify as White. Thus, the intervention has differential effects for Blacks and Whites. In Figure 3, we show the results of the intervention by racial/ethnic group, again presenting the ATT.

The effect of reducing isolation appears similar across racial/ethnic groups, and differences across groups are not substantial. Still, it is worth highlighting that older Black individuals who live in the United States commonly experience worse cognitive function than White individuals. Therefore, reducing isolation for Black individuals would be more insulative than for Whites and Latinx. This makes older Black individuals an especially vulnerable subpopulation, especially as wealth disparities mean that they cannot rely on the same economic resources as Whites.

**Figure 4.**
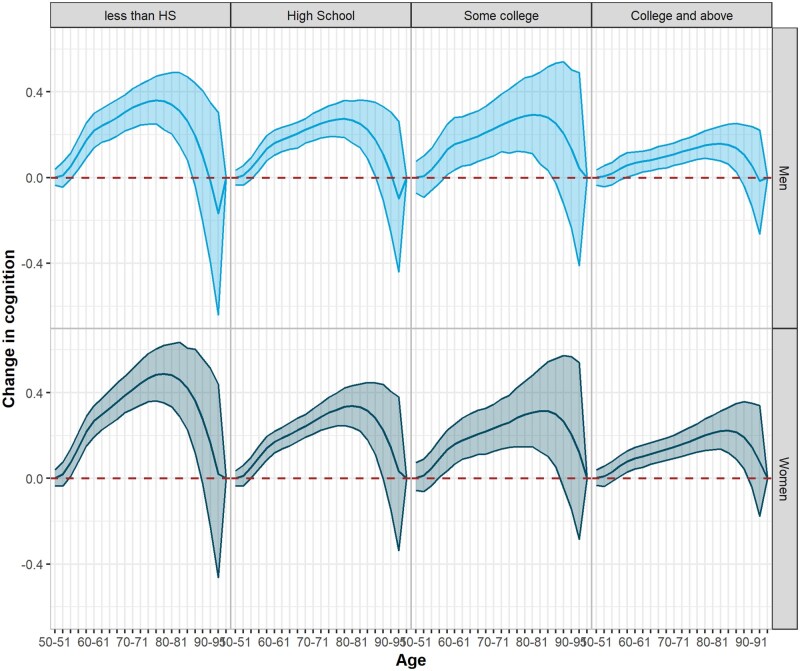
Average treatment effect on the treated showing the effect of reducing social isolation on trajectories of cognitive function across age by level of education.

A similar line of reasoning is also suggested by the results of the moderation analysis of the effect of reducing social isolation on trajectories of cognitive function by level of education ­(Figure 4). Also in this case, the overall effect of reducing social isolation is positive, with a larger effect at lower levels of education, in which individuals of racial minorities are overrepresented.

#### Mediation analysis: loneliness

We perform mediation analyses to determine the extent to which loneliness acts as a mechanism through which social isolation impacts cognitive function. Our analysis reveals that loneliness accounts for only 6% of the effect of social isolation on cognitive function—8% for men and 5% for women. This indicates that the influence of social isolation on cognitive decline is only slightly attributable to feelings of loneliness. These results suggest that, while loneliness does contribute to the relationship between social isolation and cognitive decline, the majority of social isolation’s impact on cognitive function is driven by factors other than loneliness.

#### Dynamic intervention for those living alone

Building on our previous findings, we conducted further analysis, focusing on an intervention targeted at individuals living alone. In this scenario, we added a dynamic element to the intervention to test the possible effects of reducing social isolation among those who live alone, which is a time-varying characteristic. As noted above, our goal extends beyond simply clarifying how social isolation affects cognitive function; we recognize that public health interventions seldom lead to fully exposed or unexposed populations. Therefore, we aim to understand the impact of social isolation on cognitive function through a statistical intervention that is feasible for policy implementation and minimizes spillover effects.

**Figure 5.**
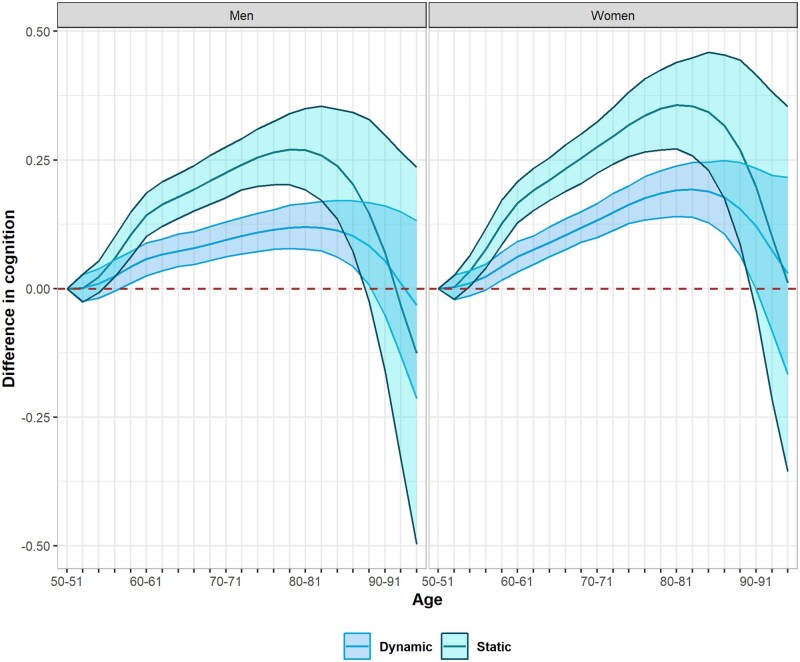
Difference in the average treatment effect on the treated for cognitive function across age, comparing the static and dynamic interventions for men and women.

We find that the protective effect against cognitive decline attributed to the dynamic intervention (intervening only on those living alone) amounts to about 50% of the TE observed when social isolation is reduced across the whole population (Figure 5). In other words, targeting the smaller subpopulation of those living alone (∼20% of the observations) accounts for 50% of the protective effect. It is important to note that the total protective effect produced by the dynamic intervention is still averaged across the entire population. This implies that when we apply the dynamic intervention across all isolated individuals living alone, the averaged effect is larger than the impact from intervening on all isolated individuals (i.e., disregarding household composition).

## Limitations

The limitations in this study fall into three categories: (1) data constraints, (2) measurement constraints, and (3) methodological constraints (see also a full discussion of model assumptions in Supplementary Methods, see online supplementary material).

### Data

The initial data challenge is accessing individuals who are isolated. It is likely that those who are the most isolated may not be well represented in survey datasets. Additional insights could be gained by investigating social isolation and loneliness through qualitative research, with older adults recruited through preexisting community outreach programs. Another unavoidable aspect of using the HRS is that we cannot know for how many years this person has been isolated or lonely. This is particularly concerning if the eventual cognitive toll is related to a lifetime’s accumulation. Nevertheless, this analysis suggests even a later-life intervention appears to be insulative against cognitive decline.

### Measurement

Limitations include the measurement of social factors and the cognitive function assessment, as well as unmeasured third variables, any of which could lead to biased estimates. The binary measure of loneliness raises concerns of bias in self-reporting, for example, men and individuals with lower levels of education are less likely to report being lonely on a binary question. However, the binary indicator is highly correlated with more nuanced measurements, and we believe the benefit gained from biennial questioning over a long period outweighs the limitations.

The SII that we created includes multiple domains of isolation, following the literature (Berkman & Syme, 1979; Hong et al., 2023); however, it has not been validated. The factors we include are objective, which should reduce the risk of self-report bias, and we use two different constructions of the index.

Because we use a continuous measure of cognitive function and a self-report on loneliness and depression (both from the CES-D), we are unable to include respondents who require proxy informants. This is likely to give downwardly biased estimates of the association between social isolation, loneliness, and cognitive function. Although selection is an issue, an advantage of the long follow-up (2004–2018) is that we retain respondents from age 50 who participate in at least two waves; also, using over 130,000 person-waves reduces the bias likely to affect those most disadvantaged.

Based on evidence that the link between social isolation and cognitive function is not domain-specific for either predictor or outcome (Evans et al., 2019), we do not separate the cognitive or social domains. However, evidence from causal inference models that there may be domain-specific effects on other health and well-being outcomes (Hong et al., 2023) suggests further exploration could be fruitful in future studies.

Three elements of our approach reduce measurement concerns: (1) our primary exposure of social isolation and outcome of cognitive function are both objectively and consistently measured across the waves; (2) we include a number of covariates from across the life course; and (3) we implement a longitudinal, counterfactual design.

#### Method

We offer a full discussion of the assumptions of causal inference models (Greenland & Robins, 2009), including positivity, consistency, and exchangeability, in Supplementary Methods (see online supplementary material). Another concern in previous research on social isolation, loneliness, and health is related to reverse causality (Evans et al., 2019). For example, research shows that those who are lonely and/or isolated have less salubrious health behaviors (e.g., sleep disturbances, lower physical activity) (Hong et al., 2023; Kobayashi & Steptoe, 2018). This makes it unclear to what extent it is the social isolation/loneliness and to what extent the health behaviors that negatively impact cognitive outcomes. Again, the counterfactual design helps mitigate these concerns, but future research could benefit from analyzing other datasets that include more detailed measures of social engagement.

## Discussion

Meta-analyses have yielded mixed findings for the association between social isolation, loneliness, and cognitive function, both from cross-sectional and longitudinal studies (Evans et al., 2019; Holt-Lunstad et al., 2015). This article embeds within a life-course perspective a causal inference model that analyzes how social isolation affects later-life cognitive trajectories, independently of and through self-reported loneliness, and net of birth cohort, interview region, race/ethnicity, childhood SES, educational attainment, partnership status, household composition, labor force participation, comorbidities, and self-reported depression.

We have three key findings: (1) there is a protective effect against later-life cognitive decline of reducing social isolation; (2) it is similarly protective (though there are some minor differences) for women and men, for Whites, Blacks, and Latinx, and regardless of educational attainment; and (3) only 6% of the effect of social isolation on cognitive function operates through loneliness.

Policy implications include that because social isolation has an independent effect on cognitive function, focusing only on interventions to manage loneliness, for example, cognitive behavioral therapy (National Academies of Sciences, 2020), will not be sufficient. Instead, social isolation and loneliness must be understood as “distinct targets for interventions” (Hong et al., 2023, p. 1), and, as such, policymakers must continue to invest resources in the reduction (or prevention; Kim & Hwang, 2024)) of isolation amongst older adults. These findings highlight the need for investigation into additional mechanisms that may explain the link between social isolation and cognitive health, pointing toward a multifaceted approach to understanding and mitigating the risks associated with social isolation.

We also modelled an intervention focused on older adults who live alone. We find a differential effect. This highlights the feasibility and potential efficacy of targeted interventions, even if their overall effect size may be modest. Such interventions can play an important role in public health strategies, particularly in efficiently utilizing resources to address issues like social isolation, which have broad and complex precursors and consequences. Relatedly, public health practitioners must evaluate the viability of interventions to reduce social isolation. For example, while requiring grandchild care is clearly an inappropriate intervention, viable interventions may include improving accessibility to reduce barriers to social integration.

Finally, that the modelled intervention was dynamic emphasizes the importance of nuanced policy measures that account for the fluctuating nature of social conditions such as living alone. It suggests that interventions tailored to specific living conditions or demographic changes can yield meaningful benefits, helping insulate older individuals against cognitive decline. More generally, these findings contribute to the discourse on the design and implementation of public health interventions. They suggest that addressing social isolation—and, by extension, its effects on health—requires both a broad understanding of its impacts on the general population and a nuanced approach to targeting interventions where they can be most effective.

## Supplementary Material

gbaf254_Supplementary_Data

## Data Availability

Data are publicly available from https://hrs.isr.umich.edu/ and https://g2aging.org/home. Code will be made available upon request.
