# Supplementary material for: Disentangling social isolation, loneliness, and later-life cognitive function for older adults in the United States: evidence from causal inference modeling
Source: J Gerontol B Psychol Sci Soc Sci. 2025 Dec 16;81(3):gbaf254. doi: 10.1093/geronb/gbaf254 (PMC13376962; doi:10.1093/geronb/gbaf254)
Supplement: gbaf254_Supplementary_Data [file gbaf254_supplementary_data.zip › 08-Jan-2026_111803_Supplement_Data.zip!/JGSS suppl Hale, Lorenti, & Cunningham.docx]

***The Journals of Gerontology, Series B: Psychological Sciences and Social Sciences* Supplementary Material: Hale, Lorenti, & Cunningham: Disentangling social isolation, loneliness, and later-life cognitive function for older adults in the United States: Evidence from causal inference modeling.**

# **Methods**

This section elaborates on the methods section in the manuscript. Based on the literature cited in the manuscript, we construct a causal DAG (Figure 1), which portrays the interrelationships among the factors. To address potential feedback between cognitive function, social isolation, loneliness, and other time-varying characteristics, we use a cross-lagged design through which we model variables at time t+1 as a function of time-fixed and varying variables at time t. The temporal ordering helps minimize reverse causality-related bias. However, if there are some variables affecting other variables at the same timepoint, some of these effects may be lost, leading to effect attenuation.

We use the design described in the DAG to estimate parametric multivariable models for the time-varying variables. We apply binomial and multinomial logistic regression models for categorical variables and linear regression models for continuous variables. From the estimated models, we derive predicted probabilities and conditional expectations that are used as parameters to simulate the value of the corresponding variable for each individual at each timepoint, drawing from binomial, multinomial, and normal distributions. This procedure is repeated 500 times, by resampling the analytical sample to generate non-parametric bootstrap estimates for confidence intervals. To minimize Monte Carlo variability, we replicate the simulation 50 times within each bootstrap. The simulated data that resembles the observed data is called the natural course, as it reflects the concept of an individual following a “natural course of events” (see Supplemental Figures 1-3). Note that, because we use a synthetic cohort approach and because of the bootstrap, we do not use survey design weights; however, we have included the demographic and regional variables on which HRS bases its weights (HRS Staff 2008; Ofstedal and Weir 2011).

We then simulate the data as in the previous step, but model reducing social isolation. That is, for individuals who were isolated in the natural course, we deterministically fix the value of the time-varying variable describing the individual trajectory of social isolation to be “lower isolation.” Such a change also will affect the distribution of the other time-varying variables that are linked to social isolation in a causal chain; such a dynamic effect allows us to understand how cognition is affected by the absence of the isolation trajectory exposure.

Finally, comparing the natural course with the statistical intervention scenario (Wang & Arah, 2015), we calculate the total effect on cognitive function of the intervention of reducing social isolation.

In causal inference terms: we estimate the Total Effect (TE) of a simulated intervention comparing the outcomes under two distinct levels of social isolation. Our estimand is defined as:$TE=E\left[ Y_{x}- Y_{x*} \right],$ where $Y_{x} \mathrm{and} Y_{x*}$are the potential cognitive function outcomes under two alternative social isolation scenarios (e.g., isolated vs less-isolated). This means that in the counterfactual scenario we fix the treatment to the value corresponding with being less isolated for all individuals who were isolated in the observed data – the contrast between observed and simulated scenario defines our estimand (Lundberg et al., 2021). For the estimation of the empirical analog we refer to Table 2 in Wang and Arah, 2015.

We then split the TE of social isolation into two components: the total direct effect (TDE) and the pure indirect effect (PIE) as defined in Table 1 in Wang and Arah, 2015. The TDE is calculated by keeping the mediator’s level as it would naturally occur without any intervention on social isolation, essentially isolating the direct impact of the intervention on the outcome. On the other hand, the PIE quantifies the portion of the intervention’s effect on the outcome that is transmitted via the mediator. It is calculated as the difference between the TE and the TDE. In our analysis, the TDE represents the influence of social isolation on cognitive function that does not involve loneliness, whereas the PIE captures the effect of social isolation on cognitive function that occurs through the influence of loneliness.

# **Model Assumptions**

Like any empirical research, our study’s interpretations rely on a foundational set of assumptions. These include the correct specification of our model alongside three crucial assumptions for causal inference: positivity, consistency, and exchangeability (Greenland and Robins 2009). To satisfy the positivity assumption, each individual in the study must have a greater than zero probability of receiving the intervention. This means our dataset should not contain any groups (strata) defined by covariates where individuals are either exclusively treated or untreated. This assumption ensures that the intervention is feasible for all participants under study conditions. In our context, we hypothesize the reduction of social isolation, presupposing that it is feasible for individuals to transition from being more isolated to less isolated. However, if there are individuals for whom isolation cannot be addressed by our intervention, such a change is impossible, the resulting population-averaged effect should be smaller. This potential problem, however, should not affect the treatment effect on the treated, as they are those who actually receive the treatment. Nevertheless, as our intervention is to move individuals from “Isolated” (SII=6 to 8) to “Less isolated” (SII=0 to 5) versus to eliminate all indicators of isolation, it is reasonable to expect the intervention is feasible.

Our statistical intervention reduces social isolation among individuals identified through their isolation status. The intervention’s impact is inferred from the experiences of those not isolated; the differences in isolation status between individuals vary - from family ties to community integration. This approach underscores the intervention as an extension of naturally occurring societal interactions rather than a policy measure. This point is related to the consistency assumption, which ensures that the treatment is well-defined and that the data accurately reflects the treatment being studied. While this can be challenging for observational studies, our intervention utilizes a simplified model of social isolation that captures its natural distribution in society. Although there are diverse reasons for individuals’ isolation, we strive to ensure comparability between the isolated and less-isolated populations, and thus to ensure the exchangeability assumption, which is essential for giving our findings a causal interpretation.

The exchangeability assumption requires that we control for all the relevant confounders, such that isolated and less isolated individuals are comparable, conditional on the measured covariates, and thus we can assess the effect of social isolation on cognitive function. Despite adjusting for numerous confounders, the possibility of unobserved or imperfectly measured variables (residual confounding) remains, suggesting that while our study aims to approximate causality, it cannot fully eliminate biases. Indeed, while statistical analysis is valuable for understanding the impact of interventions aimed at reducing social isolation on cognitive function, it is crucial to consider additional factors when evaluating such interventions. To gain a deeper insight into how our statistical intervention functions, we developed a dynamic version that specifically targets individuals living alone. This approach has several potential benefits: it minimizes the risk of spillover effects, makes the intervention more feasible (or at least conceivable), and allows for a better understanding of the mechanisms through which the original intervention exerted its effects.

In sum, despite the limitations of this approach, it enables us to gain insights that are crucial for informing policy development (VanderWeele & Hernan, 2013).

# **Tables**

**Supplementary Table 1.** Full descriptive table of the analytical sample by binary Social Isolation Index score, Health and Retirement Study 2004-2018

| **Variable** | **Social Isolation Index** | | | | **Sample Size**  **(person-wave)** |
| --- | --- | --- | --- | --- | --- |
|  | **Less Isolated** | | **More Isolated** | |  |
|  | ***n*** | ***%*** | ***n*** | ***%*** | ***n*** |
| Loneliness |  |  |  |  |  |
| Not Lonely | 84240 | 74 | 29809 | 26 | 114089 |
| Lonely | 10777 | 46 | 12877 | 55 | 23604 |
| Total | 95017 | 69 | 42636 | 31 | 137653 |
| Age (mean) | 65.3 |  | 71.5 |  |  |
| Race/Ethnicity |  |  |  |  |  |
| White | 64633 | 71 | 26923 | 29 | 91556 |
| Black | 16389 | 66 | 8257 | 34 | 24646 |
| Latinx | 10705 | 63 | 6280 | 37 | 16985 |
| Other | 3290 | 74 | 1176 | 26 | 4466 |
| Gender |  |  |  |  |  |
| Men | 40377 | 71 | 16410 | 29 | 56787 |
| Women | 54640 | 68 | 26226 | 32 | 80866 |
| Childhood Socioeconomic Status |  |  |  |  |  |
| Poor | 25734 | 62 | 15533 | 38 | 41267 |
| Average | 61253 | 71 | 24648 | 29 | 85901 |
| Wealthier | 8030 | 77 | 2455 | 23 | 10485 |
| Education |  |  |  |  |  |
| Less than high school/GED | 23945 | 55 | 19351 | 45 | 43296 |
| High school | 38123 | 69 | 17275 | 31 | 55398 |
| Some College/Associate’s degree | 6256 | 79 | 1625 | 21 | 7881 |
| College+ | 26693 | 86 | 4385 | 14 | 31078 |
| Wealth Quintile |  |  |  |  |  |
| Lowest | 22920 | 52 | 20946 | 48 | 43866 |
| Second | 21483 | 67 | 10546 | 33 | 32029 |
| Third | 19319 | 79 | 5152 | 21 | 24471 |
| Fourth | 22001 | 83 | 4630 | 17 | 26631 |
| Highest | 9294 | 87 | 1362 | 13 | 10656 |
| Household composition | |  |  |  |  |
| Live alone | 14756 | 44 | 18439 | 56 | 33195 |
| With partner | 48416 | 83 | 9647 | 17 | 58063 |
| With children | 4653 | 48 | 5058 | 52 | 9711 |
| With partner and children | 14066 | 85 | 2466 | 15 | 16532 |
| Other | 13126 | 65 | 7026 | 35 | 20152 |
| Labor Force Status |  |  |  |  |  |
| Full-time/part-time work | 35394 | 84 | 6575 | 16 | 41969 |
| Retired (or partly) | 50541 | 62 | 30837 | 38 | 81378 |
| Unemployed | 2391 | 76 | 766 | 24 | 3157 |
| Not in the labor force and Disabled | 6691 | 60 | 4458 | 40 | 11149 |
| Comorbidities |  |  |  |  |  |
| Comorbidities 0 | 32821 | 79 | 8571 | 21 | 41392 |
| Comorbidities 1 | 35662 | 71 | 14375 | 29 | 50037 |
| Comorbidities 2 + | 26534 | 57 | 19690 | 43 | 46224 |
| Depressed |  |  |  |  |  |
| Yes | 9386 | 48 | 10053 | 52 | 19439 |
| No | 85631 | 72 | 32583 | 28 | 118214 |
| Region of Interview |  |  |  |  |  |
| Northeast | 14068 | 68 | 6743 | 32 | 20811 |
| Midwest | 22155 | 70 | 9509 | 30 | 31664 |
| South | 39024 | 68 | 18191 | 32 | 57215 |
| West | 19582 | 71 | 8102 | 29 | 27684 |
| Other | 188 | 67 | 91 | 33 | 279 |
| Cohort^a^ |  |  |  |  |  |
| AHEAD | 2201 | 32 | 4774 | 68 | 6975 |
| Children of the Depression Era | 6648 | 51 | 6423 | 49 | 13071 |
| Health and Retirement Study | 27126 | 65 | 14813 | 35 | 41939 |
| War-babies | 14580 | 77 | 4322 | 23 | 18902 |
| Early Baby-boomers | 19841 | 76 | 6128 | 24 | 25969 |
| Mid Baby-boomers | 16534 | 79 | 4421 | 21 | 20955 |
| Late Baby-boomers | 7574 | 81 | 1726 | 19 | 9300 |
| Early Generation X | 513 | 95 | 29 | 5 | 542 |

Notes. GED = General Equivalency Degree.

^a^ Cohorts: Asset and Health Dynamics Among the Oldest Old (AHEAD): 1919-23; Children of the Depression Era: 1924-30; Health and Retirement Study: 1931-41, War-babies: 1942-47; Early Baby-boomers: 1948-53; Mid Baby-boomers: 1954-59; Late Baby-boomers: 1960-65; and Early Generation X: 1966-71.

# **Figures**

**Supplementary Figure 1.** A comparison of the natural course with the observed data for men and women, Health and Retirement Study 2004-2018

**Supplementary Figure 2.** A comparison of the simulated data (the natural course) with the observed data with 95% confidence intervals for key time-varying variables cognitive function, isolation, loneliness, household composition, comorbidities, depression, and labor force status for women, Health and Retirement Study 2004-2018

**Supplementary Figure 3*.*** A comparison of the simulated data (the natural course) with the observed data with 95% confidence intervals for key time-varying variables cognitive function, isolation, loneliness, household composition, comorbidities, depression, and labor force status for men, Health and Retirement Study 2004-2018

# **References**

Greenland, S., Robins, J.M., 2009. Identifiability, exchangeability and confounding revisited. *Epidemiologic Perspectives & Innovations 6*, 4. https://doi.org/10.1186/1742-5573-6-4

Lundberg, I., Johnson, R., Stewart, B.M., 2021. What is your estimand? Defining the target quantity connects statistical evidence to theory. *Am Sociol Rev 86*, 532-565. https://doi.org/10.1177/00031224211004187

Petersen, M.L., Porter, K.E., Gruber, S., Wang, Y., Van Der Laan, M.J., 2012. Diagnosing and responding to violations in the positivity assumption. *Stat Methods Med Res 21*, 31-54. https://doi.org/10.1177/0962280210386207

Rehkopf, D.H., Glymour, M.M., Osypuk, T.L., 2016. The consistency assumption for causal inference in social epidemiology: when a rose is not a rose. *Curr Epidemiol Rep 3*, 63-71. https://doi.org/10.1007/s40471-016-0069-5

VanderWeele, T.J., Hernan, M.A., 2013. Causal inference under multiple versions of treatment. *J Causal Inference 1*, 1-20. https://doi.org/10.1515/jci-2012-0002

Wang, A., Arah, O.A., 2015. G-computation demonstration in causal mediation analysis. *Eur J Epidemiol 30*, 1119-1127. https://doi.org/10.1007/s10654-015-0100-z
